# MicroRNA-138 Regulates Hypoxia-Induced Endothelial Cell Dysfunction By Targeting S100A1

**DOI:** 10.1371/journal.pone.0078684

**Published:** 2013-11-11

**Authors:** Anagha Sen, Shumei Ren, Carolin Lerchenmüller, Jianxin Sun, Norbert Weiss, Patrick Most, Karsten Peppel

**Affiliations:** 1 Center for Translational Medicine, Jefferson Medical College, Philadelphia, Pennsylvania, United States of America; 2 Laboratory for Molecular and Translational Cardiology, Department of Internal Medicine III, University of Heidelberg, Heidelberg, Germany; 3 Center for Vascular Medicine, Department of Medicine III, University Hospital Carl Gustav Carus, Technical University Dresden, Dresden, Germany; Thomas Jefferson University, United States of America

## Abstract

The Ca^2+^ sensor S100A1 is essential for proper endothelial cell (EC) nitric oxide (NO) synthase (eNOS) activation. S100A1 levels are greatly reduced in primary human microvascular ECs subjected to hypoxia, rendering them dysfunctional. However mechanisms that regulate S100A1 levels in ECs are unknown. Here we show that ECs transfected with a S100A1–3′ untranslated region (UTR) luciferase reporter construct display significantly reduced gene expression when subjected to low oxygen levels or chemical hypoxia. Bioinformatic analysis suggested that microRNA -138 (MiR-138) could target the 3′UTR of S100A1. Patients with critical limb ischemia (CLI) or mice subjected to femoral artery resection (FAR) displayed increased MiR-138 levels and decreased S100A1 protein expression. Consistent with this finding, hypoxia greatly increased MiR-138 levels in ECs, but not in skeletal muscle C2C12 myoblasts or differentiated myotubes or primary human vascular smooth muscle cells. Transfection of a MiR-138 mimic into ECs reduced S100A1–3 ‘UTR reporter gene expression, while transfection of an anti MiR-138 prevented the hypoxia-induced downregulation of the reporter gene. Deletion of the 22 nucleotide putative MiR-138 target site abolished the hypoxia-induced loss of reporter gene expression. Knockdown of Hif1-α mediated by siRNA prevented loss of hypoxia-induced reporter gene expression. Conversely, specific activation of Hif1-α by a selective prolyl-hydroxylase inhibitor (IOX2) reduced reporter gene expression even in the absence of hypoxia. Finally, primary ECs transfected with a MiR-138 mimic displayed reduced tube formation when plated onto Matrigel matrix and expressed less NO when stimulated with VEGF. These effects were reversed by gene transfer of S100A1 using recombinant adenovirus. We conclude that hypoxia-induced MiR-138 is an essential mediator of EC dysfunction via its ability to target the 3′UTR of S100A1.

## Introduction

Lack of agonist-induced eNOS activation leads to endothelial dysfunction and predisposes to a host of cardiovascular pathologies [1]. We have recently identified the small EF-hand Ca^2+^ binding protein S100A1 as part of an important molecular toolkit that relays intracellular Ca^2+^ oscillations and regulates vascular tone. Patients with critical limb ischemia (CLI) are known to have impaired vasodilatory responses [2], and present with greatly reduced levels of S100A1 in ischemic muscle tissue [3]. We were able to show that S100A1 directly interacts with eNOS, prevents PKC-mediated phosphorylation of the inhibitory Thr-495 site on eNOS and augments eNOS enzymatic activity in ECs [3]. Loss of S100A1 attenuates induction of angiogenesis in response to acute tissue ischemia, thereby preventing relief of tissue malperfusion. Thus mice genetically deficient in S100A1 suffer limb loss when subjected to femoral artery resection (FAR) [3]. S100A1 levels in ECs are rapidly lost upon exposure to hypoxia and this correlates with a loss of stimulus-induced NO production [3]. Given the importance of S100A1 to the regulation of eNOS activity and overall EC physiology, we set out to investigate ischemia-induced mechanisms that regulate S100A1 expression levels in ECs. Rapid downregulation of S100A1 specifically in ECs suggested existence of post-transcriptional regulatory mechanisms, possibly involving microRNAs (MiRs). MiRs are short, ≈22 nucleotide long regulatory RNAs that suppress gene expression by binding to the 3′ untranslated region (3′UTR) of target mRNAs, inhibiting their translation and/or augmenting mRNA degradation [4]. Here we report that MiR-138 targets the 3′UTR of S100A1 and regulates its expression in a hypoxia-dependent manner specifically in ECs.

## Methods

### Ethics Statement

Collection of patient biopsy specimen: Patients gave their written informed consent to participate in the study, as described [3]. The study protocol had been approved by the ethics committee of the Medical Faculty at the Technische Universität Dresden.

Animal experiments: All experiments were performed according to protocols approved by the Institutional Animal Care And Use Committee (IACUC) of Thomas Jefferson University and complied with the Guide for the Care and Use of Laboratory Animals.

### Mice

C57Bl/6 (WT) mice were purchased from Jackson Labs (Bar Harbor, ME stock # 664).

### Femoral Artery Resection (FAR)

Induction of acute limb ischemia by FAR was done as we described earlier [3]. Briefly, mice were anesthetized with avertin, and about 10 mm of the right common femoral artery distal of the inguinal ligament was resected. All branches were carefully cauterized. Care was taken to prevent nerve damage.

### Patient Muscle Biopsy Samples

Characteristics of patients with CLI or control have been described in detail [3].

### Cell Culture

EA.hy926 endothelial cells ATCC (CRL-2922) were cultured in DMEM (4.5 g/L glucose) supplemented with 10% FBS. Human microvascular endothelial cells (HMVEC) were purchased from Lonza (CC-7030) at passage 4–5 and cultured in ATCC vascular cell basal medium (PCS-100-030) supplemented with the ATCC endothelial cell VEGF growth kit (PCS-100-041). Human vascular smooth muscle cells were purchased from the ATCC (PCS-100-012) (Manassas, VA) and cultured in ATCC vascular cell basal medium, supplemented with ATCC vascular smooth muscle cell growth kit (PCS-100-042).

For induction of gas hypoxia cells were subjected for 24 h to 1% O_2_ using a BioSpherix hypoxia chamber and ProOx C21 oxygen controller. For chemical hypoxia cells were treated for 24 h with either CoCl_2_ (250 µmol/L) or Desferroxamine (Des, 100 µmol/L) or the specific prolyl hydroxylase-2 (PHD2) inhibitor IOX2, (N- [[1, 2- dihydro- 4- hydroxy- 2- oxo- 1- (phenylmethyl)- 3- quinolinyl]carbonyl]- glycine, Cayman Chemical) [5] at 10 µmol/L. Viability of EA.hy926 ECs or primary human ECs was not compromised at CoCl_2_ concentrations less than 1 mmol/L and Desferroxamine less than 250 µmol/L for 24 h (not shown). Murine C2C12 myoblast cells (ATCC, cat # CRL-1772) were cultured in DMEM supplemented with 10% FBS 1% Penicillin/Streptomycin. Upon reaching confluency, cells were induced to differentiate into myotubes by incubation for 5 days in 2% horse serum, followed by treatment with CoCl_2_ (250 µmol/L) for 24 h before extract preparation.

### Plasmid

All luciferase reporter constructs were purchased from SwitchGear Genomics (Menlo Park, CA). The 3′UTR reporter construct of S100A1 gene (cat # S801348) comprised the 3′UTR of the human S100A1 gene cloned downstream of a constitutive ribosomal protein L10 (RPL10) promoter and renilla luciferase gene. The hypoxia reporter plasmid of the P4HA2 gene promoter (cat # S721928) was used to confirm the induction of hypoxia. 3′UTR control plasmid (cat # S804753) with SV40 T antigen 3′UTR and β-actin control plasmid (cat # S717678) with β-actin promoter were used as controls for transfection efficiency. A 3′UTR control reporter having the same promoter (RPL10) but linked to the SV40 T antigen 3′UTR gave high expression, that did not decrease upon treatment with gas or chemical hypoxia, while a hypoxia reporter construct that employed the P4HA2 promoter and SV40-3′UTR displayed about 4.8 (±1.2) fold induction upon exposure to CoCl_2_ (not shown). A gene block (IDT) comprising the entire S100A1 3′UTR, but lacking specifically the 22 nucleotide putative MiR-138 target site, was subcloned NheI to XhoI into the luciferase reporter vector to generate the ΔMiR-138 construct. All constructs were verified by sequencing using Jefferson’s genomics facility.

### Transfection

EA.hy926 ECs were cultured in 24 well plates in DMEM with 10% FBS. Cells were transfected with 1 µg of luciferase reporter constructs mixed with Lipofectamine 2000 in serum-free DMEM, according to the manufacturer’s instruction (Life Technologies). Transfection media was changed after 2–3 hours and replaced with DMEM,10% FBS. Cells were harvested 40 h after transfection. Primary HMVEC cells were transfected in DMEM with 20 nmoles microRNA mimic (Dharmacon ThermoFisher, cat # C-300605-05-0005) or Control mimic (cat # CP-004500-01-05) and media was changed after 1–2 hours. Hif1-α siRNA was purchased from SantaCruz (Dallas, TX, cat # SC-35561). To inhibit activity of microRNA-138, 40 nmoles of a hairpin antimir (Dharmacon ThermoFisher cat # IH-300605-0005) to MiR-138 was transfected into EA.hy926 cells using Lipofectamine 2000 (Invitrogen). Alternatively, in some experiments we used a cholesterol-conjugated antagomir-138 (1 µmol/L) to inhibit MiR-138 [6].

### Real Time PCR for MiR-138 Expression in Mice

RNA was isolated from mice as well as cell samples using RNAzol RT according to manufacturer’s instruction (MRC, Inc cat# RN190). 1 µg of input RNA was used to reverse transcribe cDNA using Origene (Rockville, MD) kit (cat # HP100042). Human miR-138 real time PCR primer pair (cat # HP300151) was from Origene which consisted of a universal primer- 5′-CTCTATGCGTCTGTACAAG-3′ and microRNA primer 5′-AGCUGGUGUUGUGAAUCAGGCCG-3′. Quantitative real time PCR was performed using BioRad (Hercules,CA) SYBR Green master mix and a Stratagene (La Jolla, CA) real time PCR cycler. Expression of U6 RNA was used to normalize the expression of miR-138. Characteristics of patients with CLI or control have been described. [3] RNA was isolated from these patient’s gastrocnemius muscle samples using RNAzol. qPCR for miR-138, snoRD44 and snoRD47 was done using ABM (Applied Biological Materials, Inc, Richmond, BC Canada) primers MPH01156, MPH00003 and MPH00004, respectively using manufacturer’s instruction.

### Luciferase Assays

Light Switch luciferase assay reagents (cat # LS100) were from SwitchGear Genomics. ECs were cultured and transfected in 24 well plates. To measure luciferase expression, 100 µl of assay reagents were added to each well and enzyme activity was measured, according to the manufacturer’s instruction (SwitchGear Genomics) using a Wallac Victor^3^ Multilabel counter (Perkin Elmer, MA). Luciferase expression is given as relative light units (RLU). All luciferase experiments were done in triplicate and repeated a minimum of 3 times.

### Immunoblot Assay

Cell samples were lysed in Laemmli buffer and 30 µg/Lane whole cell extract was loaded on 4–20% Tris-Glycine gel, (Life Technologies, CA) and transferred to nitrocellulose membrane. Membranes were probed for S100A1 (Acris, San Diego, CA, cat # SP5355P, β-actin (SantaCruz cat # sc-8432), Hif1-α (SantaCruz cat# sc-10790) total eNOS (BD Biosciences cat# 610297) or phospho Thr-495 eNOS (BD Biosciences cat# 612707). Protein expression was quantitatively assessed using an Odyssey scanner (Li-Cor Biosciences, Lincoln NE). All immunoblot assays were done in duplicate and repeated a minimum of three times.

### Matrigel Tube Formation Assay

Growth-factor reduced Matrigel Matrix (BD Biosciences, cat# 356231) was diluted 1∶1 with ATCC vascular cell basal medium (no additives). HMVEC (approx 200,000 cells) were transfected with MiR-138 mimic or scramble control. 48 h later cells were infected with recombinant Adenovirus expressing S100A1 and GFP from a bicistronic insert [7], or control at an multiplicity of infection (MOI) of 17. After 24 h cells were detached from the plastic support with trypsin and seeded onto the Matrigel matrix in medium consisting of 9 parts ATCC medium and 1 part ATCC medium containing ATCC endothelial cell VEGF growth kit (PCS-100-041). Images were taken 24 h later and digitized using NIH-ImageJ software. Original images of the matrigel tube formation assays are included as online supplement Figure S3.

### Nitric Oxide Assay

HMVEC (approx 100,000 cells) were transfected with MiR-138 mimic or scramble control. 48 h later cells were infected with recombinant Adenovirus expressing S100A1 and GFP from a bicistronic insert, or control at a MOI of 17. 8 h later the infection medium was removed and cells were starved overnight in ATCC vascular cell basal medium supplemented with 0.2% FBS. NO production was then stimulated with the addition of 50 ng/ml VEGF (R&D systems). Medium was collected 24 h later and NO levels were measured using a fluorescent NO/Nitrite/Nitrate assay (Cayman Chemical, cat# 780051) according to manufacturer’s instructions.

### Statistical Analysis

One-way ANOVA with Tukey posttest for multiple comparisons was used to analyze the appropriate data using GraphPad PRISM software. Data are shown as ± SEM in the figures. A *P* value of <0.05 was considered statistically significant. All experiments were done independently a minimum of three times. Each set-up was done at least in duplicate for each repetition.

## Results

### The 3′UTR of the S100A1 mRNA Regulates S100A1 Gene Expression

**Figure 1:**
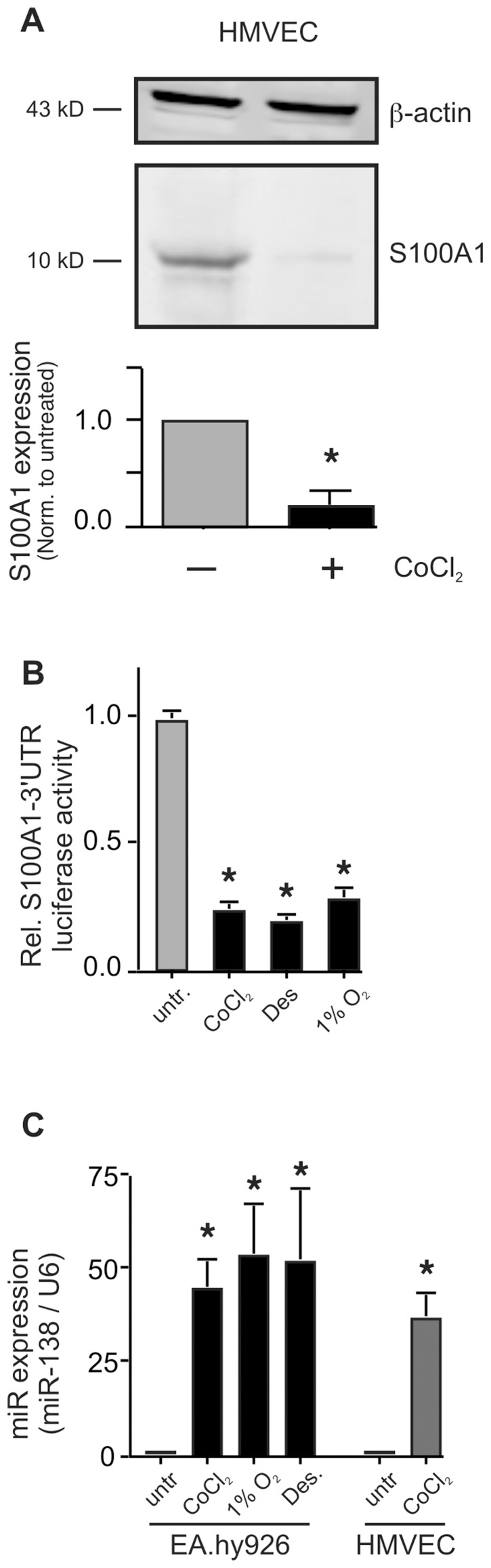
Hypoxia reduces S100A1 gene expression in Endothelial cells via its -3′UTR. A) Primary human microvascular endothelial cells (HMVECs) were subjected to chemical hypoxia by treatment with CoCl_2_ (250 µmol/L) for 24 h before extract preparation and immunoblot analysis. Expression levels of S100A1 were quantitated using a LICOR Odyssey near infrared scanner and normalized to those of β-actin. The experiment was done 4 times, each in duplicate. *, P<0.05 vs normoxic. B) EA.hy926 ECs were transfected with the S100A1–3′UTR luciferase reporter and subjected to chemical (CoCl_2_ or Desferroxamine (Des) or gas hypoxia (1% O_2_), for 24 h. Luciferase expression was measured as described in the Methods section and presented as relative light units (RLU) normalized to untreated cells. *, P<0.02 vs untreated normoxic. The experiment was performed 4 times, each in triplicate. Expression of a luciferase reporter linked to a control 3′UTR (from SV40 T antigen) was not changed (not shown). C) Expression of MiR-138 was measured by qPCR in EA.hy926 ECs or primary HMVECs treated with 1% O_2_ or CoCl_2_ (250 µmol/L) or Desferroxamine (Des, 100 µmol/L) for 24 h. Levels of the small nuclear RNA U6 were assessed in parallel and used to normalize expression. The experiment was done 4 times, each in triplicate. *, P<0.01 vs untreated. U6 expression did not change during hypoxia.

The rapid ischemia-induced downregulation of S100A1 that we observed in both human and mouse ECs [3], suggested possible involvement of post-transcriptional regulatory mechanisms. S100A1 protein expression in primary human microvascular ECs is drastically repressed by chemical hypoxia (CoCl_2_, 250 µmol/L, Figure 1A), the rapidity of which suggested involvement of post-transcriptional mechanisms, possibly involving micro-RNAs (MiRs). MiRs typically bind to the 3′UTR of target mRNAs, repressing their translation or leading to their degradation [4]. In order to specifically examine the role of the 3′UTR in the potential regulation of S100A1 protein expression, we assessed expression of a S100A1-3′UTR luciferase reporter gene in human EA.hy926 ECs, subjected to low oxygen (1%O_2_) or chemical hypoxia (CoCl_2_ or Desferroxamine, Des). Reporter gene expression was reduced to about 25% of that observed in normoxic cells, regardless whether chemical hypoxia or low oxygen was used (Figure 1B). A control reporter gene having the same promoter (from the ribosomal protein 10) but linked to the SV-40-3′UTR gave high expression, that did not decrease upon treatment with low oxygen or chemical hypoxia (data not shown). In addition, EA.hy926 ECs transfected with a hypoxia reporter construct that employed the P4HA2 promoter and SV-40-3′UTR displayed about 4.8 (±1.2) fold induction upon exposure to CoCl_2_ (data not shown). Reduction of S100A1–3′UTR reporter gene expression was dose dependent for treatment with both CoCl_2_ and Desferroxamine (Des, Figure S1). Importantly, viability of the EA.hy926 was not compromised at concentrations of CoCl_2_ less than 1 mmol/L and of Desferroxamine less than 250 µmol/L for 24 h (not shown).

### MiR-138 Targets the S100A1-3′UTR in ECs

Regulation of gene expression by the 3′UTR suggested possible involvement of a regulatory microRNA [4]. The 3′UTR of S100A1 is relatively short (196 bases for the human and 186 bases for the murine isoform). Since both human and murine S100A1 genes appear subject to the same degree of hypoxia-induced downregulation [3], we reasoned it likely that a conserved micro RNA would target a species-conserved region of the 3′UTR. Using MiRanda (at microrna.org) [8] we conducted a database search for conserved micro RNAs that would target both the human and mouse S100A1 3′UTR. The only micro RNA that registered a “hit” for S100A1–3′UTR of both species was microRNA–138 (MiR-138). This micro RNA is encoded by two different intergenic loci on chromosomes 3 and 16 for hsa-MiR-138-1 and hsa-MiR-138-2 respectively (mouse chromosomes 9 and 8 resp.). Deep sequencing reads overwhelmingly detect only the 5p segment of both MiR-138-1 and MiR-138-2 [9], [10]. The mature MiR-138 is conserved between human and mouse and alignment of the human and mouse S100A1–3′UTRs with MiR-138 shows the binding site of this micro RNA to be in the most conserved region of the 3′UTR (95% identical) whereas the rest of the S100A1-3′UTR shares ∼70% identity between the two species (Figure S2). Indeed hypoxia (induced by either low oxygen or chemical reagents) drastically increased expression of MiR-138 in both EA.hy926 as well as primary microvascular ECs (HMVEC, Figure 1C).

**Figure 2:**
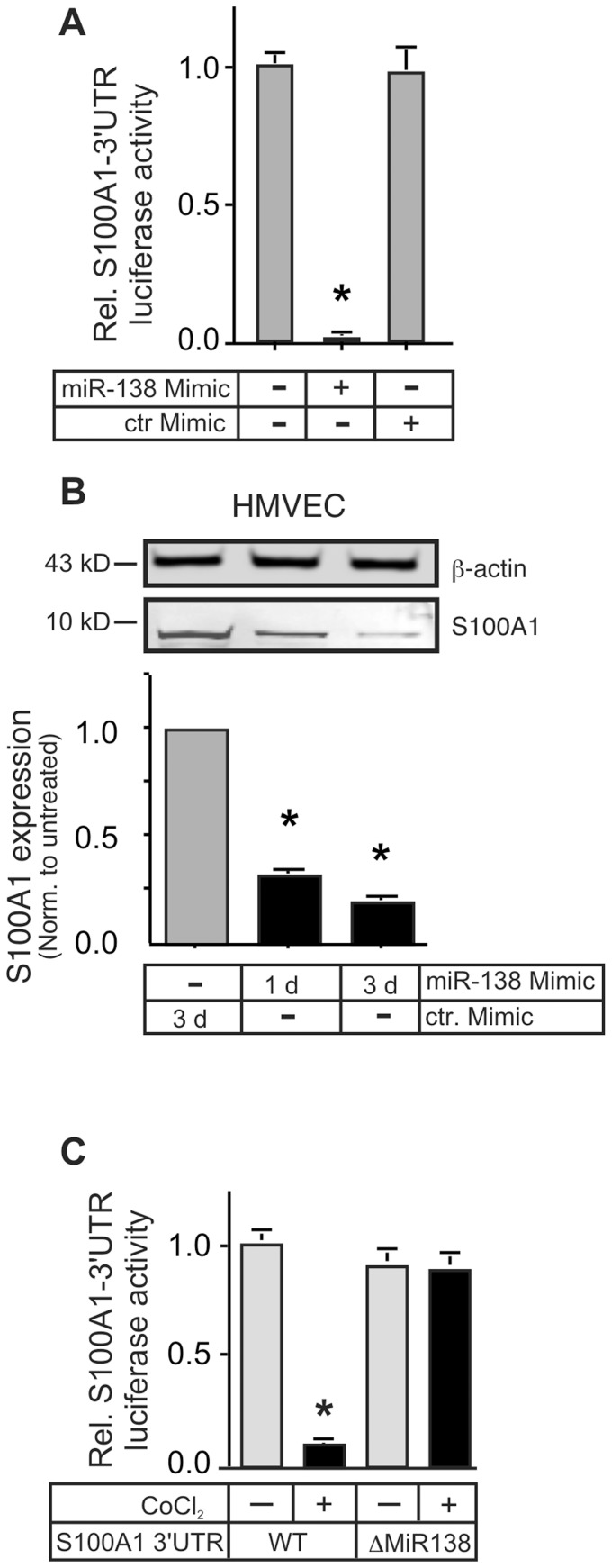
MiR-138 decreases S100A1 gene expression in Endothelial Cells. A) EA.hy926 ECs were co-transfected with the S100A1–3′UTR luciferase reporter gene and either a MiR-138 mimic or scrambled control mimic. Luciferase activity in cell lysates was measured 24 h later and is reported as relative light units (RLU) normalized to untreated cells. *, P<0.01 vs ctr. mimic. The experiment was done 3 times, each in triplicate. Expression of a luciferase reporter linked to the control 3′UTR was not changed by the mimic treatment (not shown). B) Primary HMVEC were transfected with the MiR-138 mimic for 1 or 3 days or control mimic for 3 days. Cell extracts were immunoblotted for S100A1 or β-actin (as loading control). Transfection with the control mimic did not change S100A1 levels compared to untransfected HMVECs (not shown). A representative immunoblot is shown. The experiment was performed 3 times. Expression of S100A1 was normalized to β-actin. *, P<0.05 vs ctr. Mimic. C) EA.hy926 ECs were transfected with either the wild-type (WT) S100A1–3′UTR luciferase reporter gene or a S100A1–3′UTR with deletion of the 22 nucleotide putative MiR-138 target site (ΔMiR138). 24 h later cells were subjected to chemical hypoxia (250 µmol/L CoCl_2_). Luciferase activity in cell lysates was reported as relative light units (RLU) normalized to WT untreated cells. *, P<0.01 vs untreated. The experiment was done 3 times, each in triplicate.

In order to verify the potential targeting of S100A1 by MiR-138, we co-transfected a MiR-138 mimic (Dharmacon) together with the S100A1–3′UTR reporter into EA.hy926 cells. The MiR-138 mimic reduced reporter gene expression by over 95% after 24 h (Figure 2A). When transfected into primary HMVECs the mimic significantly reduced S100A1 protein levels (Figure 2B), reminiscent of that achieved by chemical hypoxia (Figure 1A), while transfection with a scrambled control mimic did not change reporter gene expression. Confirmation of the role of MiR-138 in the regulation of S100A1 was obtained by deleting the putative 22 nucleotide MiR-138 target site within the S100A1–3′UTR of the luciferase reporter. This construct (ΔMiR-138), in all other elements identical to the wild-type (WT) S100A1–3′UTR reporter, showed no reduction of gene expression when transfected into EA.hy926 cells subjected to chemical hypoxia (Figure 2C).

**Figure 3:**
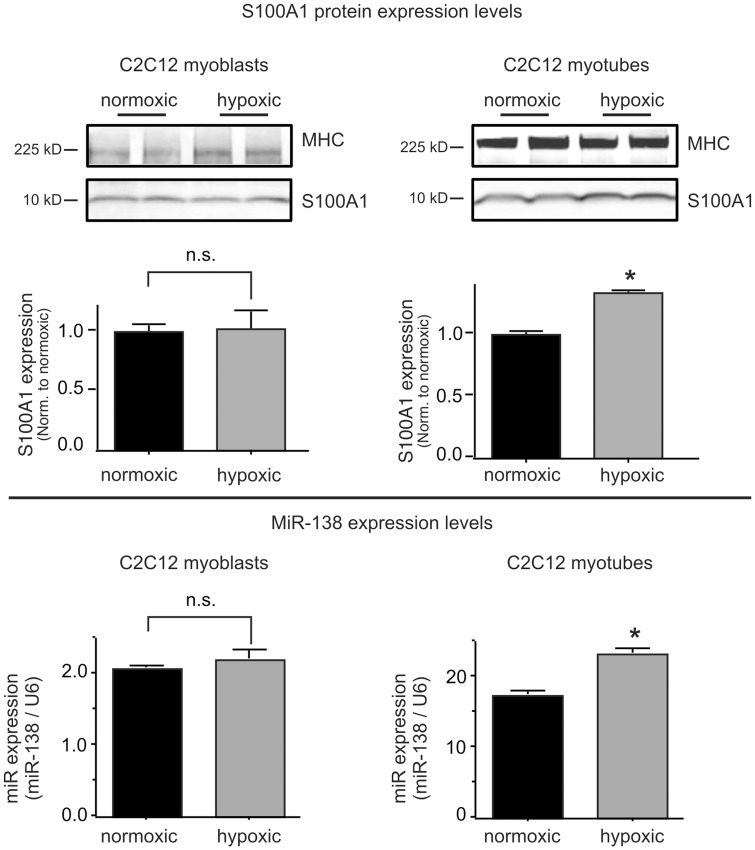
Hypoxia does not increase MiR-138 in skeletal muscle cells. Murine C2C12 skeletal myoblasts (left panels) or differentiated myotubes (right panels) were subjected to hypoxia for 24 h. Protein extracts were immunoblotted for S100A1 protein expression (upper panels) and expression of MiR-138 by qPCR (lower panels). Expression of Myosin Heavy Chain (MHC) was used to verify differentiation. Experiment was performed 3 times, each in duplicate. *, P<0.05 vs normoxic.

**Figure 4:**
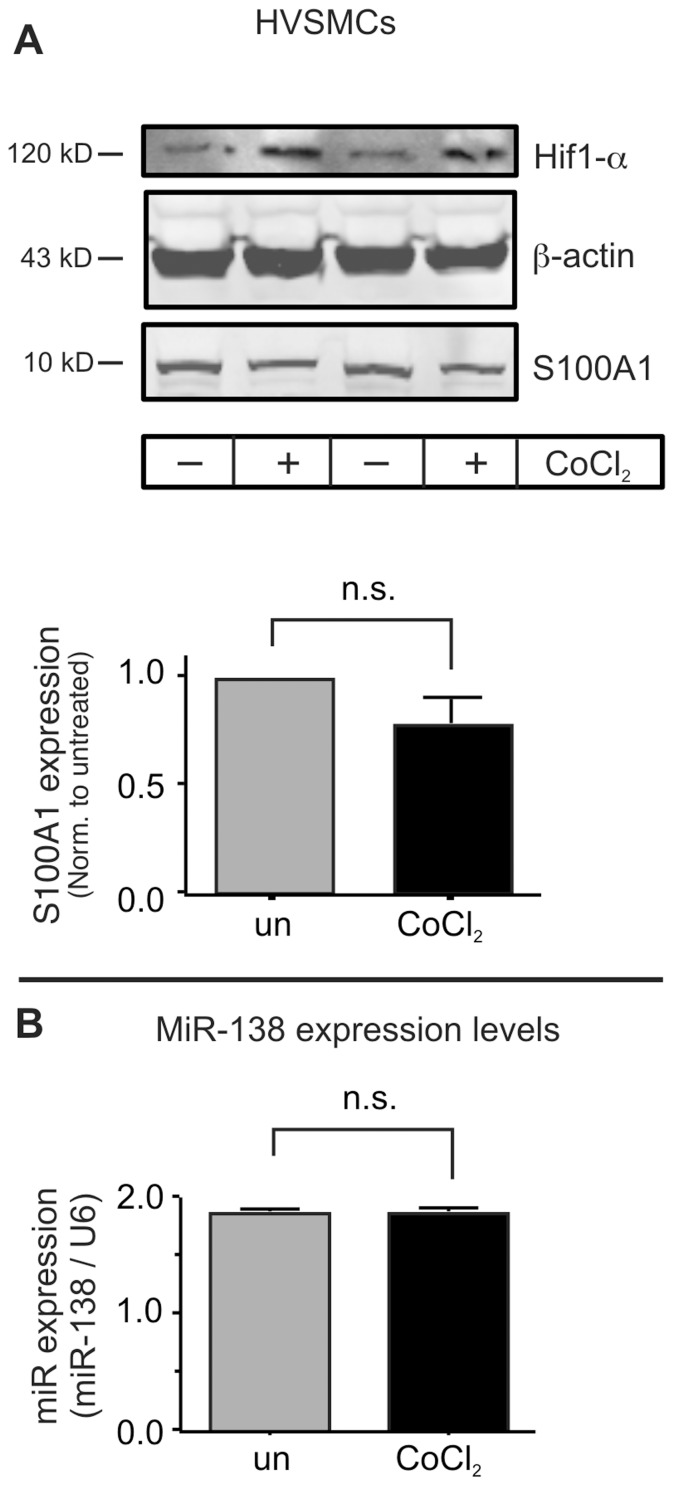
Primary human vascular smooth muscle cells do not change S100A1 nor MiR-138 levels during hypoxia. A) Primary human microvascular smooth muscle cells (HVSMCs, obtained from the ATCC) were subjected to hypoxia for 24 h. Protein extracts were immunoblotted for Hif1-α (to verify induction of hypoxia), β-actin (to verify equal loading), and S100A1. B) Expression of MiR-138 was by qPCR. Experiment was performed 3 times, each in duplicate.

We next determined whether the hypoxia-induced increase in MiR-138 was cell type selective with regard to other cell types relevant to muscle physiology. Neither skeletal muscle myoblasts, differentiated myotubes (Figure 3), nor primary human vascular smooth muscle cells (HVSMCs, Figure 4) display much of a change of S100A1 or MiR-138 expression when subjected to hypoxia, even though all of these cell types express both S100A1 and MiR-138. These findings agree with our previous study that demonstrated significant differences in hypoxia-induced attenuation of S100A1 expression between isolated murine skeletal muscle myofibers and primary ECs [3].

**Figure 5:**
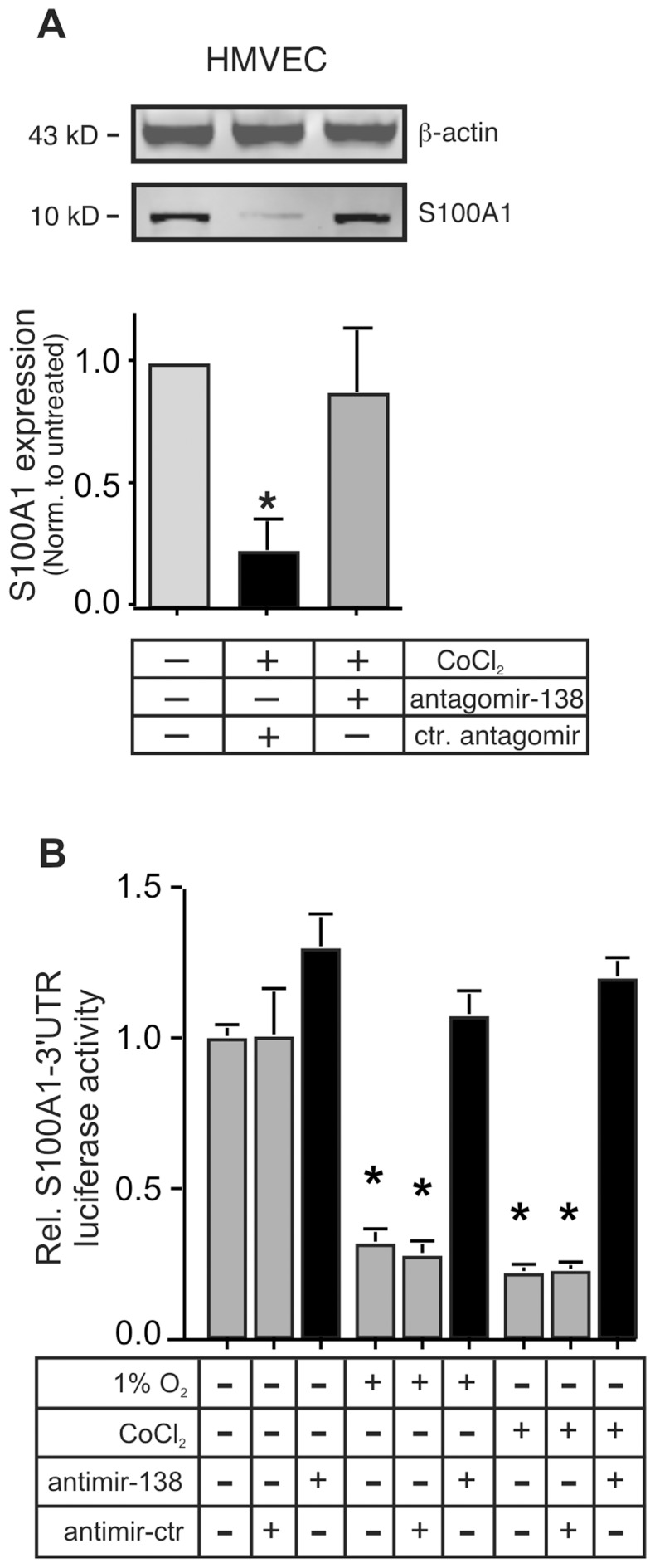
Specific inhibition of MiR-138 prevents the hypoxia-induced loss of S100A1 in ECs. A) Primary HMVECs were transfected with the antagomir-138 (or control antagomir) and subjected to chemical hypoxia (CoCl_2_, 250 µmol/L) for 24 h before extract preparation and immunoblot analysis. S100A1 expression was normalized to that of β-actin. The experiment was done 3 times, each in duplicate. *, P<0.05 vs control antagomir. B) EA.hy926 ECs were transfected with an antimir-138 (or control) and subjected to either gas hypoxia (1% O_2_) or chemical hypoxia (CoCl_2_, 250 µmol/L) for 24 h. Luciferase activity in cell lysates was reported as relative light units (RLU) normalized to untreated cells. The experiment was done 3 times, each in triplicate. *, P<0.02 vs antimir-138 treated.

In order to assess the physiological relevance of endogenously produced MiR-138, we inhibited the function of endogenously generated MiR-138 by incubating primary HMVECs with a specific antagomir to MiR-138 [6]. Transfection of the antagomir-138 completely prevented the loss of S100A1 protein expression, while a scramble control antagomir had no effect on the hypoxia-induced downregulation of S100A1 expression in HMVECs. This indicates that hypoxia regulates S100A1 expression in ECs predominantly via MiR-138 (Figure 5A). Confirmation of this was also obtained by co-transfecting a hairpin anti-MiR-138 (antimir-138, Dharmacon) into EA.hy926 ECs together with the S100A1-3′UTR reporter. The antimir-138 completely prevented the downregulation of the reporter gene induced by either low oxygen or chemical hypoxia, while co-transfection with a scrambled control antimir had no effect (Figure 5B). Together, these findings strongly suggest that hypoxia induces cell-type selective expression of MiR-138 and that this induction can drastically reduce S100A1 protein levels in ECs.

### MiR-138 is Increased in Critical Limb Ischemia

**Figure 6:**
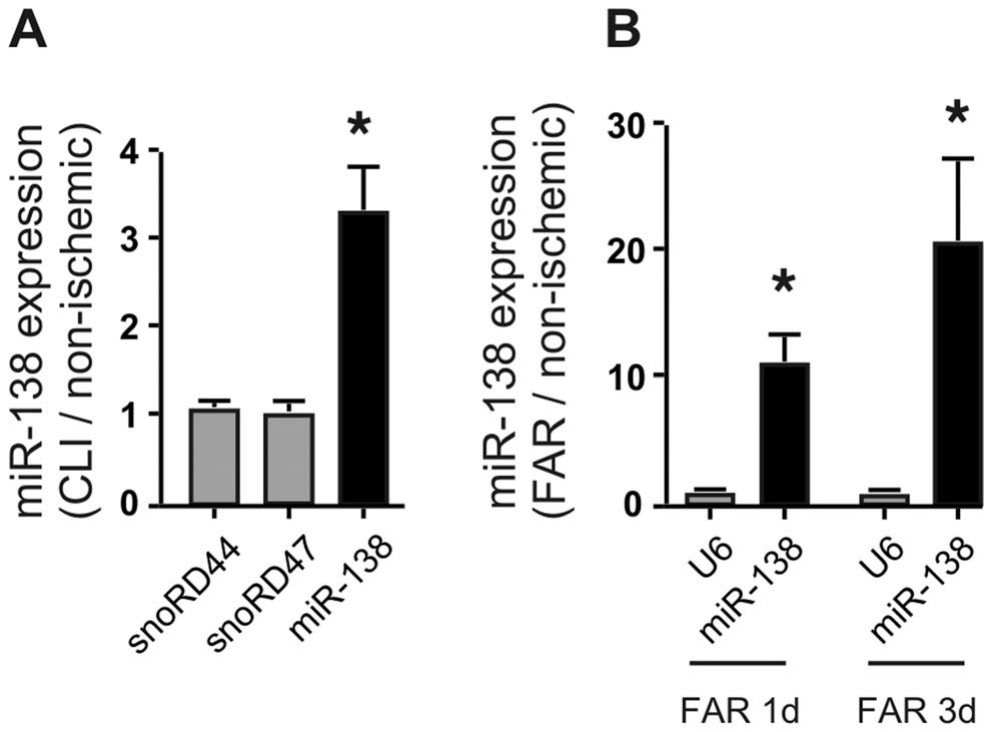
MiR-138 levels are increased in ischemic muscle tissue. A) Gastrocnemius muscle biopsy specimens from patients with CLI and non-ischemic control [3] were analyzed for expression levels of MiR-138 and the housekeeping small nucleolar RNAs snoRD44 and snoRD47 by qPCR. Expression levels are presented as fold CLI/normal. n = 4; *, P<0.05 vs snoRD44 or 47, whose expression levels in CLI samples were not significantly different from normal. B) Gastrocnemius muscle biopsy specimens from mice post femoral artery resection (FAR) and non-ischemic contralateral control were analyzed for expression levels of MiR-138 and the U6 small nuclear housekeeping RNA by qPCR at times indicated. Expression levels are presented as fold FAR/normal. n = 4; * P<0.05 vs U6, whose expression levels in FAR samples were not significantly different from normal.

We had demonstrated greatly reduced S100A1 levels in mal-perfused muscle tissue of both human patients with CLI and mice after induction of limb ischemia by FAR [3]. In support of a crucial role of MiR-138 in the regulation of endothelial S100A1 expression, we found that levels of MiR-138 were significantly increased in samples taken from both human patients with CLI (Figure 6A) and ischemic mouse gastrocnemius muscles (Figure 6B), while expression of housekeeping small nucleolar RNAs RD44 and RD47 did not change, demonstrating that tissue ischemia leads to an increase in MiR-138 that could potentially be responsible for the observed downregulation of S100A1. It is likely that the majority of the observed increase in MiR-138 under this pathological condition is attributable to the EC compartment, given that both skeletal muscle cells as well as vascular smooth muscle cells do not demonstrate a significant increase of MiR-138 when subjected to hypoxia (Figures 3, 4).

### Hif1-α Activation is Required for the Hypoxia-induced Expression of MiR-138

**Figure 7:**
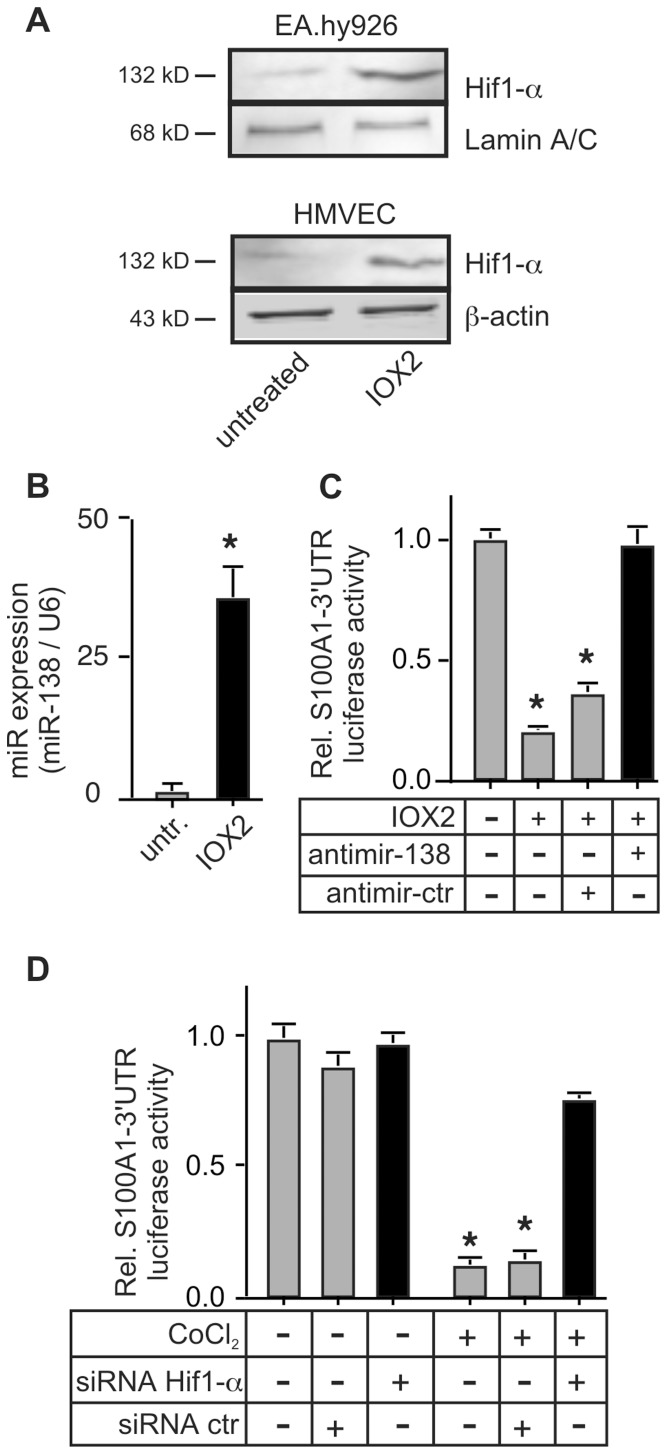
Hif-1α mediates the reduction of S100A1-3′UTR reporter gene expression. A) EA.hy926 (upper panel) or primary HMVEC (lower panel) cells were exposed to the prolyl-hydroxylase-2 inhibitor IOX2 (10 µmol/L) for 24 h to induce Hif1-α, prior to extract preparation. A representative immunoblot is shown to verify Hif1-α induction. The experiment was done 3 times with similar results. β-actin was used to control for protein loading. B) Expression levels of MiR-138 were assessed by qPCR in extracts prepared from EA.hy926 ECs subjected to 24 h treatment with 10 µmol/L IOX2. C) EA.hy926 ECs were co-transfected with the S100A1–3′UTR luciferase reporter gene and either the antimir-138 or scramble control (Dharmacon). 24 h later cells were incubated with IOX2 to induce Hif1-α stabilization. *, P<0.02 vs untreated. For both B, C, the experiment was done 3 times, each in triplicate. D) EA.hy926 ECs were transfected with the S100A1-3′UTR reporter gene and co-transfected with siRNA against Hif1-α, or control scramble siRNA. Cells were then subjected to chemical hypoxia with 250 µmol/L CoCl_2_ for 24 h before luciferase activity was assessed. *, P<0.02 vs normoxic, P<0.05 vs siRNA Hif1-α. Experiment was performed 3 times, each in triplicate.

The hypoxia-induced increase in MiR-138 led us to examine the contribution of the transcription factor hypoxia-induced factor 1-α (Hif1-α) to the regulation of MiR-138 expression. Specific inhibition of the prolyl-hydroxylase 2 enzyme by IOX2 leads to the stabilization of Hif1-α in endothelial cells [5] (Figure 7A), and greatly increased MiR-138 levels (Figure 7B), concomitant with reduced S100A1–3′UTR reporter gene expression. This was specifically due to the induction of MiR-138 since co-transfection with the antimir-138 completely reversed the decrease in reporter gene expression (Figure 7C). The critical role of Hif1-α in the induction of MiR-138 in ECs was proven by siRNA-induced silencing of Hif1-α, which completely prevented the hypoxia-induced downregulation of reporter gene activity (Figure 7D).

### MiR-138 Induces EC Dysfunction Specifically via Inhibition of S100A1

**Figure 8:**
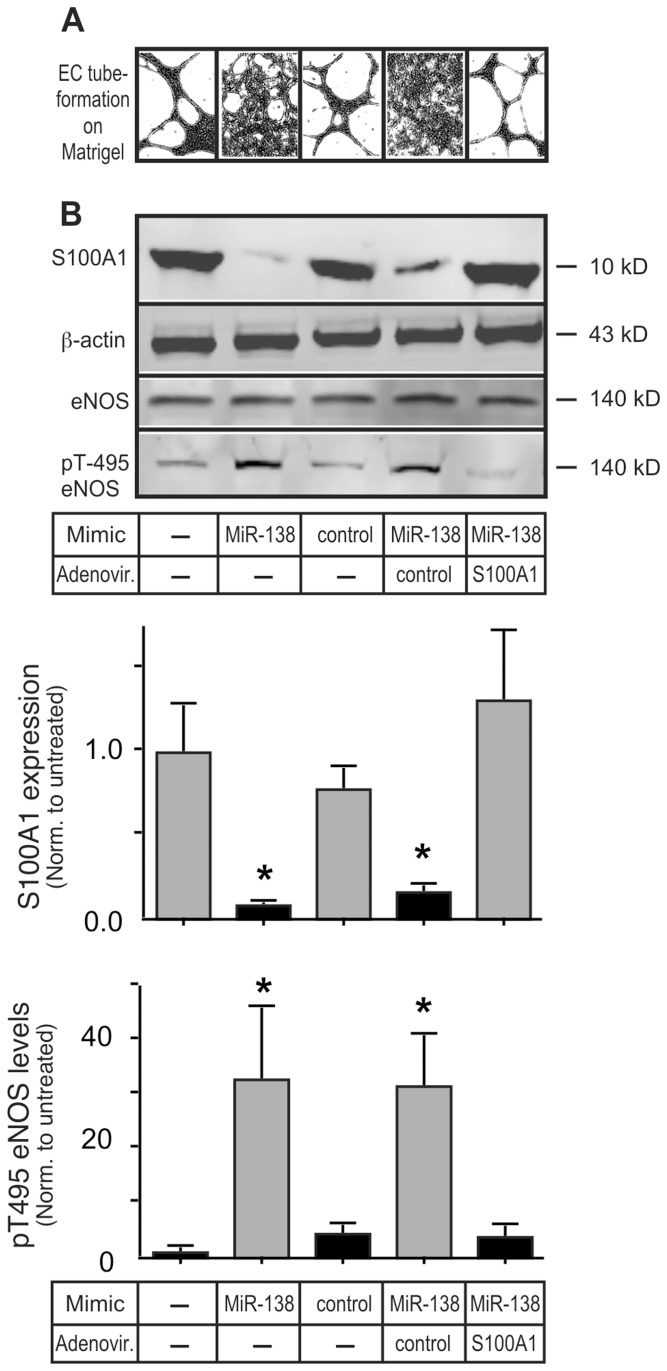
MiR-138 compromises EC Matrigel-induced capillary formation by inhibiting S100A1. A) Primary HMVEC were transfected with the MiR-138 Mimic or scramble control Mimic. 48 h later cells were infected (MOI = 17) with either control Adenovirus or Adenovirus expressing S100A1. 24 h later cells were seeded onto Matrigel matrix. Images of EC tube formation were taken 24 h later and digitized using Image J (Original pictures of EC tube formation are included as Figure S3). B) Cell extracts of HMVEC treated in parallel to those in (A) were immunoblotted for S100A1, total or p-Thr 495 eNOS, or β-actin (as loading control). Representative images are shown. The experiment was done 3 times, each in duplicate. Expression levels of S100A1 and pT-495 eNOS were normalized to β-actin and total eNOS, respectively. The experiment was done 3 times, each in duplicate. *, P<0.05 vs untreated.

**Figure 9:**
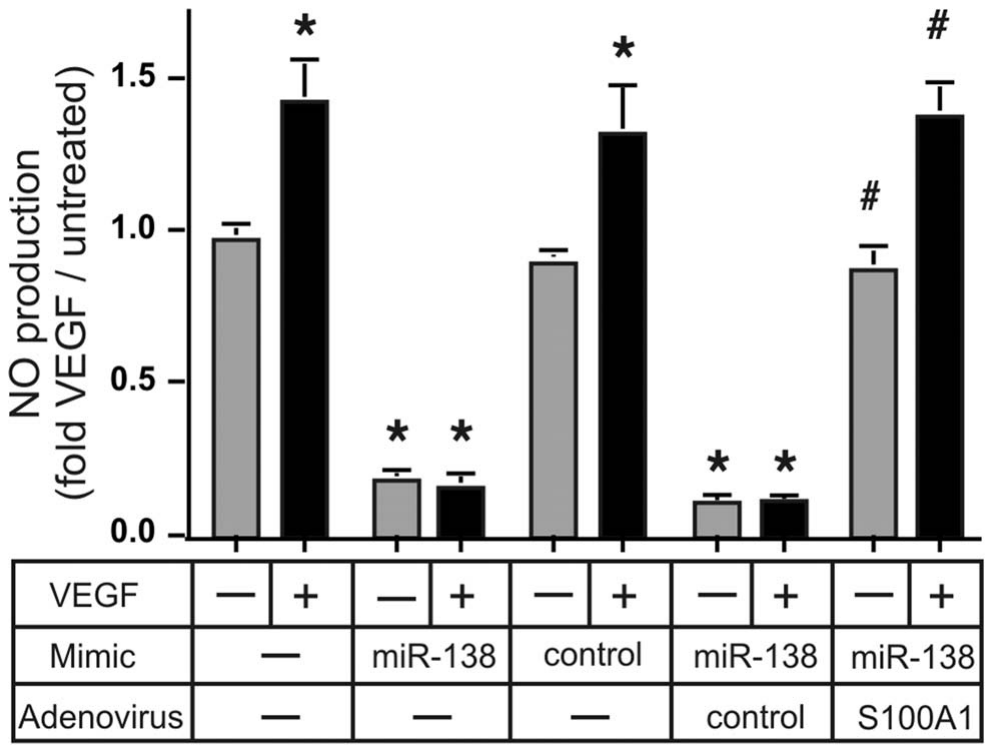
MiR-138 compromises VEGF-stimulated NO production by inhibiting S100A1. Primary HMVEC cells treated in parallel to those in Figure 8 were starved for 24 h in medium supplemented with 0.2% FBS before being treated with 50 ng/ml VEGF. Supernatants were collected 24 h later and analyzed for nitrate/nitrite levels. The experiment was done 3 times, in duplicate. *, P<0.01 vs no Mimic, #, P<0.01 vs control Adenovirus. Re-expression of S100A1 reverses the MiR-138 induced EC dysfunction.

In order to examine the physiological relevance of increased EC MiR-138 expression we transfected primary HMVEC with the MiR-138 mimic and tested the ability of these cells to form capillary-like tube networks on Matrigel matrix. Tube formation was greatly reduced in MiR-138 mimic transfected cells, coincident with greatly reduced levels of S100A1 (Figures 8A, B, see Figure S3 for the original, non-digitized, photo-micrographs). Infection with recombinant adenovirus expressing S100A1 [11] re-established S100A1 levels to near normal in MiR-138 mimic transfected cells (Figure 8B), and re-established tube formation capability (Figure 8A). We had previously demonstrated that S100A1 is an essential factor required for proper eNOS activation in ECs [3], [7]. While total eNOS levels remained unchanged in MiR-138 mimic transfected ECs, the reduction of S100A1 greatly increased eNOS phosphorylation on Thr-495, a demonstrated eNOS inhibitory site [12] (Figure 8B). This change in eNOS phosphorylation also was normalized upon re-expression of physiologic levels of S100A1, even in the continued presence of the MiR-138 mimic. Physiologically, the increased phosphorylation of Thr-495 induced by the MiR-138 mimic abolished VEGF stimulated NO production in ECs (Figure 9). Restoration of physiological levels of S100A1, even in the presence of MiR-138 mimic, normalized the VEGF-stimulated NO production (Figure 9). Both capillary network formation on matrigel matrix and stimulus induced NO formation are considered hallmarks of healthy endothelial cell function. Since NO production contributes to the pro-angiogenic actions of VEGF [13], these experiments provide conclusive evidence for the pathophysiological relevancy of increased MiR-138 expression and subsequent suppression of S100A1 in ECs.

## Discussion

Appropriate expression of S100A1 appears essential for correct intracellular signaling in large variety of different cell types. On a cellular level, S100A1 expression is highest in cardiomyocytes where this Ca^2+^- binding EF-hand protein orchestrates the complex Ca^2+^ fluxes required for optimal excitation–contraction coupling [14], however this protein is also found in significant quantities in skeletal muscle myofibers [15], neurons [16], and ECs [3], [7], amongst others. First evidence of an important contribution of S100A1 to EC physiology was gleaned from the hypertensive phenotype of S100A1 gene deficient mice, which presented with impaired endothelium-dependent vasodilation [7]. Subsequently we showed that S100A1 is an essential activator of eNOS and is required for post-natal vascular angiogenesis in response to ischemia [3]. Pathophysiologic relevance may be derived from the observation that S100A1 levels are severely downregulated [3], while MiR-138 levels are increased, in both human muscle biopsies procured from patients with CLI, as well as mice with induced limb ischemia (Figure 6). It is now clear that distinct regulatory mechanisms govern expression of S100A1 in different cell types. First evidence of this was obtained in isolated ECs and myofibers where hypoxia induces a rapid loss of S100A1 selectively in ECs [3].

**Figure 10:**
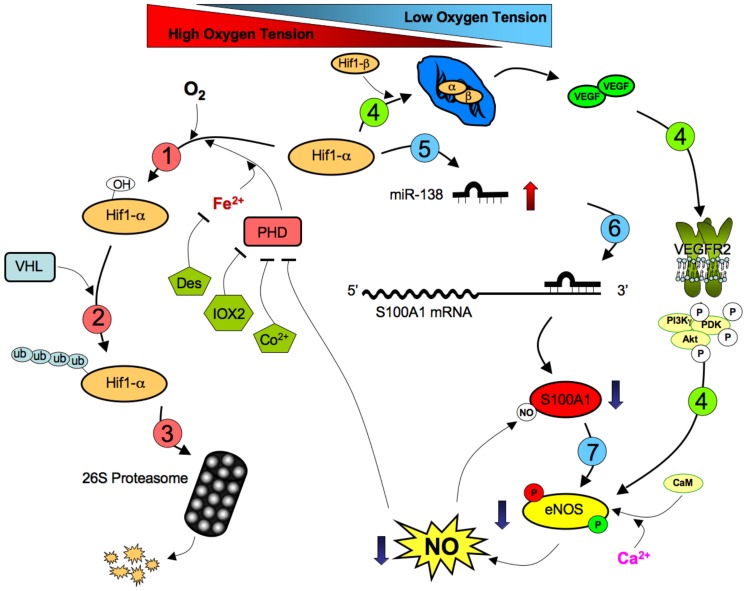
Proposed Scheme of S100A1 regulation by MiR-138. (**1**) Under normal oxygen tension, the transcription factor Hif1-α is continuously hydroxylated by the action of cellular prolyl-hydroxylases (PHDs) in a reaction that requires Fe^2+^ and O_2_ as co-factors. (**2**) Hydroxylation of Hif1-α promotes binding of the Von Hippel-Lindau (VHL)-E3 ubiquitin ligase complex, promoting poly-ubiquitination and (**3**) degradation via the 26S proteasome complex. The action of PHDs are inhibited directly by IOX2 and Cobalt and indirectly by iron chelators, such as Desferroxamine (Des) as well as low oxygen levels. (**4**) Under low oxygen tension the Hif1-α protein becomes stabilized in the nucleus and promotes transcription of the pro-angiogenic vascular endothelial growth factor (VEGF) gene. VEGF promotes activation of eNOS by signaling through VEGFR2, promoting phosphorylation of the stimulatory Ser-1177 site. Increased eNOS activity raises nitric oxide (NO) production, which inhibits PHDs, further promoting Hif1-α stabilization in a positive feed-back loop. (**5**) To maintain cellular homeostasis, stabilization of Hif1-α also promotes increased production of MiR-138, (**6**) which binds to the 3′UTR of the S100A1 mRNA, leading to drastically reduced S100A1 levels and reduction of eNOS activity by promoting phosphorylation of the inhibitory Thr-495 site (**7**), in a counterbalancing negative feed-back loop. Endothelial dysfunction develops when these carefully balanced multiple feedback loops become dysregulated allowing for prolonged MiR-138 expression with consequent loss of S100A1 and reduced eNOS activity.

Here we report, for the first time, on the mechanisms by which S100A1 expression is selectively regulated in ECs (see Figure 10 for a proposed scheme). The most important finding of our work is the identification of MiR-138 as a crucial determinant of S100A1 expression in ECs subjected to hypoxia. It is clear that MiR-138 itself is dynamically regulated in response to low oxygen levels in a cell-type specific manner. It is tempting to speculate that one or both of the MiR-138 genes may be direct targets of Hif1-α, since we have shown here that this transcription factor is indispensable for the hypoxia-induced expression of MiR-138 and the subsequent repression of S100A1. In this light it is interesting to note that MiR-138 has been reported to directly target Hif1-α in cultured cells [17], [18], potentially allowing for feed-back control of MiR-138 expression during hypoxia. However, we can not rule out other ways by which MiR-138 expression is controlled since processing of the mature MiR-138 from the pre-MiR-138 also appears to be a regulated step in some tissues [19]. Furthermore it is clear that hypoxia, *per se*, does not increase MiR-138 levels in all cells, as we have shown that C2C12 skeletal muscle cells, while expressing both MiR-138 and S100A1, do neither increase MiR-138, nor decrease S100A1 when subjected to hypoxia. While a recent report by Li et al. demonstrated a hypoxia-induced increase of MiR-138 in airway smooth muscle cells [20], we did not observe a significant hypoxia-induced change in either MiR-138 or S100A1 expression in primary human vascular smooth muscle cells, however it is likely that the significant differences in smooth muscle cell type (airway vs. microvascular) and the different species (rat in the study by Li et al. vs human in ours) are at least partly responsible for the observed differences.

MiR-138 has thus far been reported to be involved in a variety of cellular functions related to cell motility [21] and control of tumor growth [22], [23], [24], [25]. While MiR-138, like all other microRNAs, has a large number of potential targets, its suppression of S100A1 in ECs may be especially important for vascular physiology since we have shown here that restoration of S100A1 levels in ECs with increased MiR-138 is sufficient to reverse EC dysfunction, as manifest by restored stimulus-dependent NO generation and Matrigel tube formation capability.

## Conclusions

Here we show for the first time that S100A1, a central co-activator of eNOS activity, is drastically downregulated by hypoxia-induced MiR-138 in endothelial cells. Reduced eNOS activity leads to accompanying increases in vascular risk factors such as hypertension, vascular wall oxidative stress, increased platelet aggregation and reduced ischemia-promoted angiogenesis [1]. These manifestations of endothelial dysfunction are a hallmark of most chronic cardiovascular diseases including peripheral arterial disease. Patients with CLI, the most advanced form of PAD, represent a significant unmet clinical challenge with overall poor prognosis and limited options [26], [27]. As strategies to manipulate microRNA levels in vivo become more mature [6], [28], MiR-138 might represent an attractive target for the treatment of pathologies that have underlying EC dysfunction.

## Supporting Information

Figure S1 **EA.hy926 ECs were transfected with the S100A1-3′UTR reporter gene and treated for 24 h with the indicated doses of CoCl_2_ or Desferroxamine (Des).** Chemical hypoxia dose-dependently decreases S100A1 gene expression. The experiment was done in triplicate and repeated 3 times. *, P<0.05 vs untreated.
(TIF)

Figure S2 A) Alignment of miR-138 with the human S100A1-3′UTR as predicted by miRANDA. The MiR seed region is boxed in red. B) Alignment of human and mouse S100A1-3′UTRs. The predicted miR-138 target region (deleted in the DMiR138 construct) is outlined in red. Overall homology of the 3′UTR is 72%, homology of the miR-138 target is 95% (21/22).
(TIF)

Figure S3 **Original (not digitized) Matrigel tube formation images of those shown in** **Figure 8** **.**
(TIF)
