# Correction: MicroRNA-138 Regulates Hypoxia-Induced Endothelial Cell Dysfunction By Targeting S100A1

**DOI:** 10.1371/annotation/53080a85-89cc-4a84-8fd9-0eb0c19cc05d

**Published:** 2013-12-16

**Authors:** Anagha Sen, Shumei Ren, Carolin Lerchenmüller, Jianxin Sun, Norbert Weiss, Patrick Most, Karsten Peppel

An affiliation for the sixth author was incorrectly omitted from the by-line. In addition to institutions numbers 1 and 2, Patrick Most is also affiliated withe the following institution: DZHK (German Center for Cardiovascular Research), Partner site Heidelberg/Mannheim, Heidelberg University Hospital, Heidelberg University, Germany.

