# Correction: MicroRNA-138 Regulates Hypoxia-Induced Endothelial Cell Dysfunction By Targeting S100A1

**DOI:** 10.1371/annotation/c7bddd8d-9f15-45f4-a886-740b351a39b6

**Published:** 2014-01-03

**Authors:** Anagha Sen, Shumei Ren, Carolin Lerchenmüller, Jianxin Sun, Norbert Weiss, Patrick Most, Karsten Peppel

The sixth author, Patrick Most, was incorrectly indicated as having contributed equally to the article with the first author, Anagha Sen. The sixth author should instead be indicated as being a co-senior author with the last author, Karsten Peppel. 

