# Supplementary figures and images for: MicroRNA-138 Regulates Hypoxia-Induced Endothelial Cell Dysfunction By Targeting S100A1
Source: PLoS One. 2013 Nov 11;8(11):e78684. doi: 10.1371/journal.pone.0078684 (PMC3823839; doi:10.1371/journal.pone.0078684)

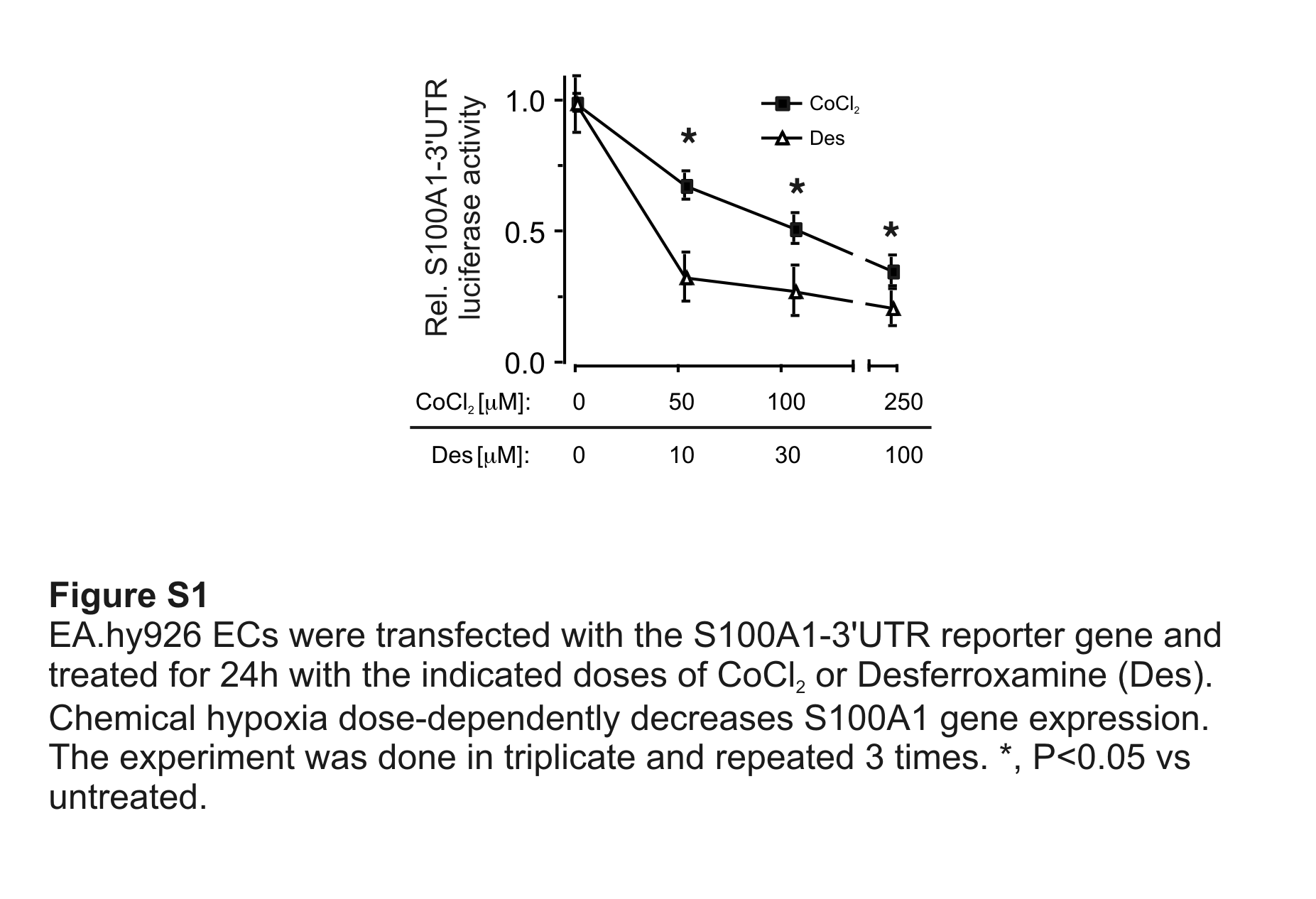

Supplement: Figure S1 — EA.hy926 ECs were transfected with the S100A1-3′UTR reporter gene and treated for 24 h with the indicated doses of CoCl2 or Desferroxamine (Des). Chemical hypoxia dose-dependently decreases S100A1 gene expression. The experiment was done in triplicate and repeated 3 times. *, P<0.05 vs untreated. (TIF) [file pone.0078684.s001.tif]

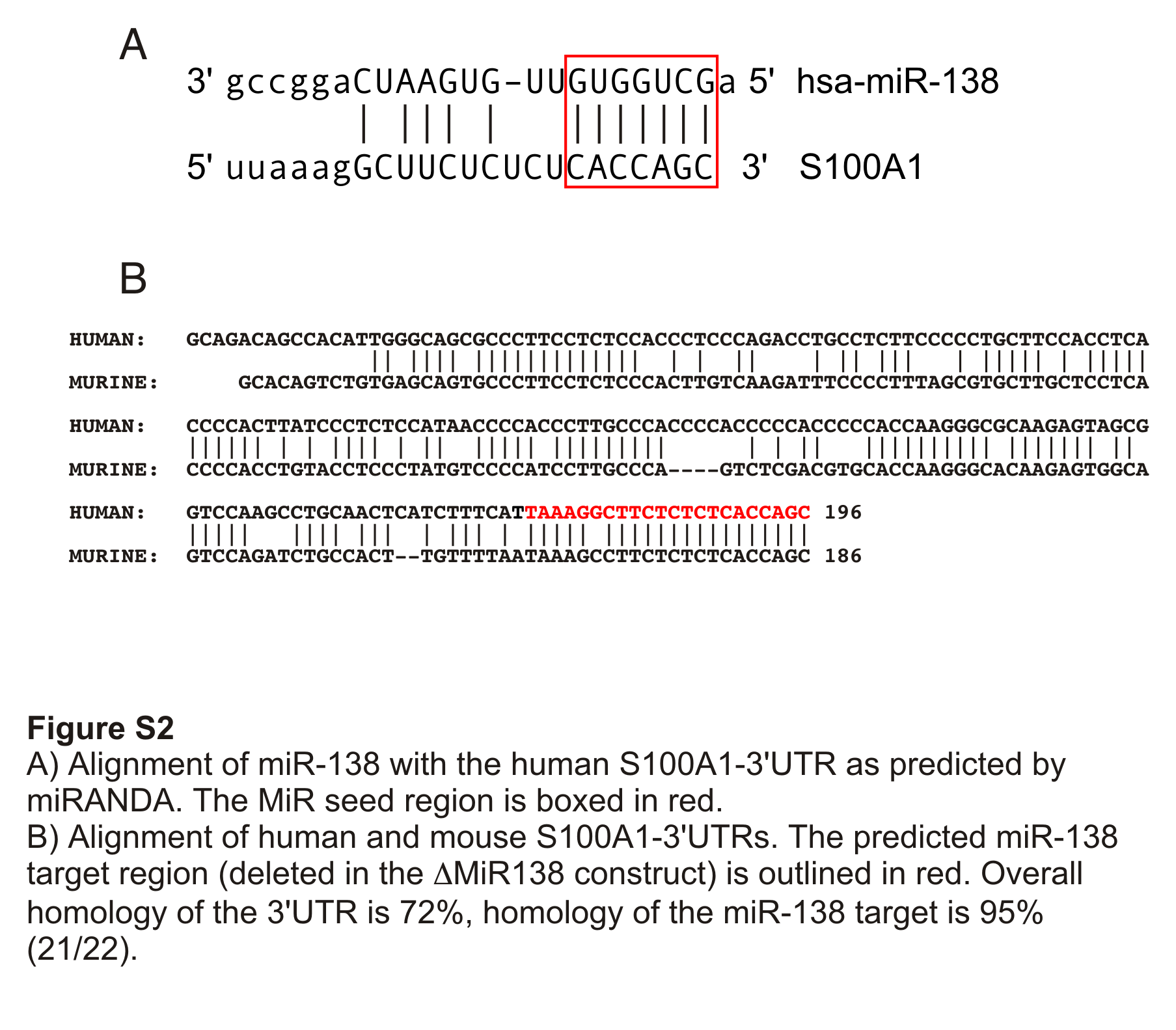

Supplement: Figure S2 — A) Alignment of miR-138 with the human S100A1-3′UTR as predicted by miRANDA. The MiR seed region is boxed in red. B) Alignment of human and mouse S100A1-3′UTRs. The predicted miR-138 target region (deleted in the DMiR138 construct) is outlined in red. Overall homology of the 3′UTR is 72%, homology of the miR-138 target is 95% (21/22). (TIF) [file pone.0078684.s002.tif]

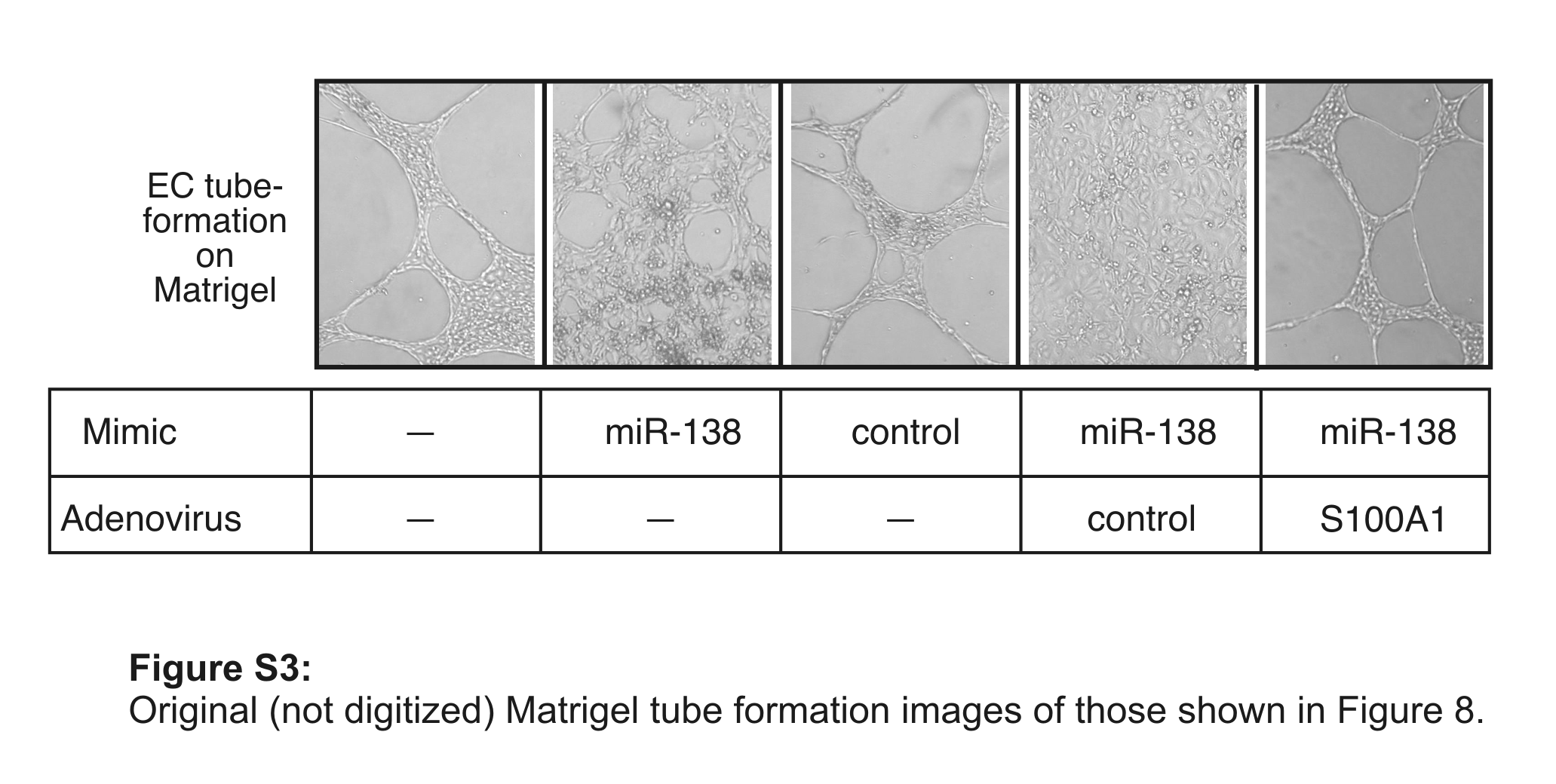

Supplement: Figure S3 — Original (not digitized) Matrigel tube formation images of those shown in Figure 8 . (TIF) [file pone.0078684.s003.tif]
